# Examining the role of protein structural dynamics in drug resistance in *Mycobacterium tuberculosis*†
†Electronic supplementary information (ESI) available. See DOI: 10.1039/c7sc03336b


**DOI:** 10.1039/c7sc03336b

**Published:** 2017-10-16

**Authors:** Daniel J. Shaw, Rachel E. Hill, Niall Simpson, Fouad S. Husseini, Kirsty Robb, Gregory M. Greetham, Michael Towrie, Anthony W. Parker, David Robinson, Jonathan D. Hirst, Paul A. Hoskisson, Neil T. Hunt

**Affiliations:** a Department of Physics , University of Strathclyde , SUPA , 107 Rottenrow East , Glasgow , G4 0NG , UK . Email: neil.hunt@strath.ac.uk; b School of Chemistry , University of Nottingham , Nottingham , UK . Email: jonathan.hirst@nottingham.ac.uk; c Strathclyde Institute of Pharmacy and Biomedical Science , University of Strathclyde , Glasgow , UK . Email: paul.hoskisson@strath.ac.uk; d STFC Central Laser Facility , Research Complex at Harwell , Rutherford Appleton Laboratory , Harwell Science and Innovation Campus , Didcot , OX110PE , Oxon , UK; e Department of Chemistry and Forensics , Nottingham Trent University , Clifton Lane , Nottingham , NG11 8NS , UK

## Abstract

Antimicrobial resistance represents a growing global health problem. The emergence of novel resistance mechanisms necessitates the development of alternative approaches to investigate the molecular fundamentals of resistance, leading ultimately to new strategies for counteracting them. To gain deeper insight into antibiotic–target interactions, the binding of the frontline anti-tuberculosis drug isoniazid (INH) to a target enzyme, InhA, from *Mycobacterium tuberculosis* was studied using ultrafast two-dimensional infrared (2D-IR) spectroscopy and molecular simulations. Comparing wild-type InhA with a series of single point mutations, it was found that binding of the INH–NAD inhibitor to susceptible forms of the enzyme increased the vibrational coupling between residues located in the Rossmann fold co-factor binding site of InhA and suppressed dynamic fluctuations of the enzyme structure. The effect correlated with biochemical assay data, being reduced in the INH-resistant S94A mutant and absent in the biochemically-inactive P193A control. Molecular dynamics simulations and calculations of inter–residue couplings indicate that the changes in coupling and dynamics are not localised to the co-factor binding site, but permeate much of the protein. We thus propose that the resistant S94A mutation circumvents subtle changes in global structural dynamics caused by INH upon binding to the wild-type enzyme that may impact upon the formation of important protein–protein complexes in the fatty acid synthase pathway of *M. tuberculosis*.

## Abstract 2

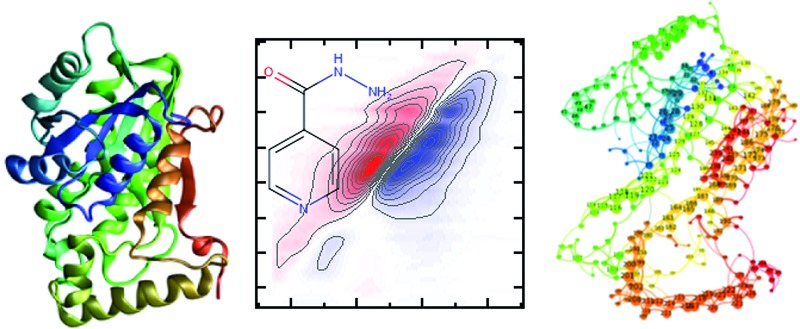
2D-IR spectroscopy reveals a role for protein structural dynamics in antimicrobial-resistance.

## Introduction

The emergence and spread of antimicrobial resistance is a global threat to human health. As a result of new resistance mechanisms, current therapeutics are being rendered ineffective and our ability to treat common infections is threatened. There is thus a clear need for the continuous development of new antimicrobials for the treatment of infections, otherwise currently routine medical procedures such as surgery and cancer chemotherapy will become impossible and costs associated with health care will increase. Our ability to develop novel intervention strategies relies on a thorough understanding of the molecular mechanisms of resistance to inform design of the next generation of drugs.

*Mycobacterium tuberculosis*, the aetiological agent of Tuberculosis (TB), exemplifies the problem of antimicrobial resistance. The World Health Organisation (WHO) estimated 10.4 million new cases of TB in 2015 with approximately 1.4 million deaths attributed to the disease. Indeed, the WHO now classifies TB as the most dangerous infectious disease after HIV.^1^ Standard treatment of drug-sensitive TB is through the use of the pro-drug isoniazid (INH) coupled with rifampicin. INH was first shown to be effective against TB in 1952 and remains a front-line pharmaceutical. The longevity of service, along with incomplete or improperly-administered treatment regimens, has contributed to the emergence of drug-resistant strains of the bacterium, such as multi-drug resistant (MDR) and extensively drug resistant (XDR) *M. tuberculosis*, which now represent a global health crisis.^1^

**Fig. 1:**
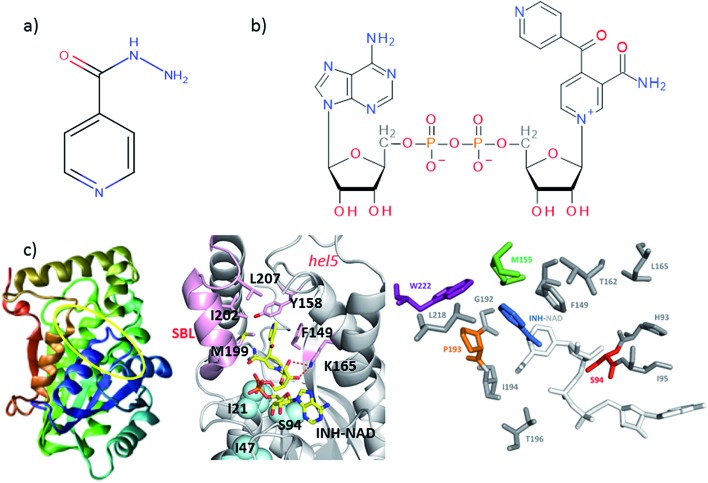
Molecular structures of (a) isoniazid (INH) and (b) the adduct molecule formed between INH and NAD that is an inhibitor of InhA.^4^,^5^ (c) Left, crystal structure of InhA (PDB ; 1zid),^5^ the color scheme runs from blue to red beginning from residue 1. The INH–NAD binding site is shown by a yellow oval. Centre diagram shows relative positions of key residues within the INH–NAD binding site. Residues clinically-implicated in resistance are shown using ball representations, other key residues referred to in the text are shown using a stick representation. Carbon atoms in the INH–NAD adduct are shown in yellow; nitrogen in blue; phosphorous in orange and oxygen in red. Right, relative spatial positions of mutated residues studied in this work to the INH–NAD adduct. S94 is shown in red, W222 in purple, P193 in orange. The NAD body of the inhibitor is shown in white, the INH-related portion is shown in blue.

Despite over 70 years of use, the in-depth molecular-level mechanism of action, and therefore resistance, is still unclear for INH.^2^–^4^ INH (Fig. 1(a), and ESI Scheme 1†) is a pro-drug, requiring activation *in vivo* by the catalase peroxidase enzyme, KatG. This creates an isonicotinoyl radical that reacts with NAD^+^ forming the INH–NAD adduct (Fig. 1(b)) that has been shown to be a slow, tight-binding inhibitor of the 2-*trans*-enoyl-reductase enzyme (InhA) that forms part of the essential FASII mycolic acid biosynthesis pathway in *M. tuberculosis*.^4^,^5^ While other interactions of INH have been identified, the FASII pathway still represents the most attractive pharmaceutically-validated target for new drug development in TB.^6^ Interestingly, much of the clinical resistance observed is *via* recessive mutations in katG, whereas dominant mutations occur in InhA.^7^ InhA is a target of both INH and the second line TB drug ethionimide, as it has been shown that the transfer of the InhA S94A mutant allele in *M. tuberculosis* is sufficient to confer resistance to both drugs.^7^ Moreover, over-expression of *inhA* also confers INH resistance in *M. tuberculosis.*^8^

A number of single point mutations of InhA have been clinically-implicated in INH resistance including I21V, I47T, and S94A. These residues appear near to the co-factor binding site and cause a reduction in binding affinity for NADH.^2^,^3^,^9^ Structural studies of InhA with both the NADH co-factor and the INH–NAD inhibitor in place have been reported^5^,^7^,^10^,^11^ and the structure of apo–InhA has been published recently.^12^ These studies show that InhA contains a Rossmann fold, a widely distributed structural feature composed of a series of alternating β-strand and α-helical segments in which the β-strands form a single parallel β-sheet through hydrogen bonding (Fig. 1(c)). However, virtually no global change to the enzyme structure is found in resistant mutations, while biochemical studies indicate that resistance is not mediated simply through reduced affinity for the INH–NAD inhibitor.^2^,^3^,^9^ Mutation has been observed to affect communication within the InhA homo-tetramer, since co-factor binding becomes cooperative.^3^ As InhA is part of a multi-enzyme complex in the cell, it is proposed that INH–NAD binding influences protein–protein interactions, either within the InhA tetramer or with other FASII proteins.^3^,^13^,^14^

These results combine to suggest that understanding the mechanism of INH resistance in *M. tuberculosis* may be informed by biophysical insight that goes beyond static structural studies. To contribute to the understanding of this problem, we have applied ultrafast 2D-IR spectroscopy alongside computational molecular simulations to probe the interaction of wild-type InhA with both the NADH co-factor and INH–NAD inhibitor. Typically, 2D-IR is applied under circumstances where a specific vibrational chromophore gives highly localised insight.^15^–^19^ These studies have revealed significant modulation of protein structural dynamics as a result of substrate or ligand binding,^20^,^21^ but extensive work on the InhA system suggests that resistance may have its roots in more global processes because binding appears to influence protein–protein interactions. To study this we explored the capability of 2D-IR to reveal changes as a result of ligand binding to an unlabeled protein by examining the amide I spectroscopy of the whole molecule. Combining this with mutational studies of the S94A mutation of InhA that is clinically-implicated in resistance, the INH-susceptible W222A and the biologically-inactive P193A control, which still retains a similar secondary structure to the wild-type,^22^ allowed deeper insight into the mechanism of action of INH with InhA (Fig. 1(c)). Examining the impact of drug binding to its target enzyme and specific mutations in this manner, we show that subtle structural and dynamic effects occur upon ligand binding; these begin in the Rossmann fold co-factor binding site but extend to remote regions of the enzyme structure. We therefore propose that this information highlights dynamic and structural communication pathways within enzymes that offers new directions to explore in the attempt to combat antimicrobial resistance.

## Experimental

### InhA preparation and enzyme assays

InhA was prepared *via* over-expression in *E. coli* and purified using methods described previously.^22^ Enzyme assay data were consistent with previous studies of InhA and have been published elsewhere.^22^ To ensure that the prepared enzymes were free of NADH prior to subsequent experiments, InhA samples were checked after purification and subsequent concentration by means of UV-vis absorption. NADH presence would be indicated by an absorption peak at 340 nm, a feature that was absent in the UV-vis spectra recorded for our samples (see Fig. S1†).

### Sample preparation for infrared spectroscopy

Purified fractions from the InhA preparation were pooled to obtain a starting enzyme concentration of ∼0.5–1 mg ml^–1^, as quantified by UV-vis absorption spectroscopy. These were concentrated *via* sequential centrifuge filtration and buffer-exchange into 200 mM deuterated sodium phosphate buffer, pD 7.4 containing trace (<0.5%) sodium dodecyl sulfate (SDS) to aid protein stabilisation at high concentration. The final sample concentration range was ∼20–30 mg ml^–1^ (0.8 mM), sufficient for all IR spectroscopy measurements. To reaffirm both structural integrity and activity, 1–2 μl of the enzyme was removed, re-diluted to ∼2 μM, and assayed.^22^ The highly concentrated InhA was also checked for consistency with low concentration samples *via* UV-vis spectroscopy. Confirming activity at 0.8 mM was challenging due to the large absorbances of the assay constituents, but when InhA was added to 150 μM NADH and 50 μM DD–CoA, an almost immediate drop to zero of the NADH absorbance was observed, indicating that the InhA was active at 0.8 mM concentration.

For all IR measurements, the samples were held between two CaF_2_ windows separated by a spacer of 50 μm thickness. Samples were finely adjusted with amounts of deuterated buffer (without additional SDS) to provide peak absorptions of 0.15–0.2 at the amide I central frequency of 1647 cm^–1^. FT-IR measurements of apo–InhA at 0.8, 0.4 and 0.27 mM concentration were made and could be scaled and overlaid to within the signal to noise level of the FT-IR instruments, providing additional confirmation that no InhA aggregation takes place under these conditions.

Apo–InhA spectra were collected at a concentration of 0.8 mM for all mutants described in the main article. To obtain the 2D-IR data for InhA with NADH present, 20 μl volumes of 0.8 mM InhA and 0.8 mM NADH were mixed and measured 45–60 minutes after mixing. InhA samples inhibited by the INH–NAD complex were prepared based on previously published procedures,^4^,^5^ but due to the extremely high NADH concentration that direct scale-up would have required, which would lead to large convolutions in the InhA amide I spectral response, they were adapted for a 1 : 1 : 1 mixture of InhA, NADH and INH all at 0.8 mM concentration. The mixture was incubated overnight at room temperature along with 1.6 mM of MnCl_2_ under N_2_ atmosphere to prevent H–D exchange. The biochemical pathway for producing INH–NAD is shown in the ESI (Scheme 1†).^5^

### Infrared spectroscopy

The method for obtaining 2D-IR spectra has been described previously.^23^ Briefly, the FT-2D-IR method was employed, which uses a sequence of three mid-IR laser pulses arranged in a pseudo pump-probe beam geometry.^24^,^25^ The pulses were generated by the ULTRA laser system consisting of a Ti:sapphire laser pumping a white-light seeded optical parametric amplifier (OPA) equipped with difference frequency mixing of the signal and idler. Mid IR pulses with a temporal duration of <100 fs; a central frequency of 1650 cm^–1^ with a bandwidth of ∼400 cm^–1^ were employed.^26^ All reported spectra were obtained using a waiting time of 250 fs and parallel pump-probe polarization direction. Each data point in the 2D-IR spectra displayed results from the averaged acquisition of 12 000 laser shots. It has been established that the direct contribution of either NADH or INH–NAD to the 2D-IR spectra was negligible at the concentrations studied and therefore any changes reported are due to the effect of ligand binding on the enzyme.^27^,^28^

### Computational modelling

Four different variants of InhA from *M. tuberculosis* were studied using MD simulations and network models of the one exciton Hamiltonian in order to investigate the effect of mutation and ligand binding on the structure and dynamics of the protein. The four variants chosen were the wild-type enzyme in complex with NADH (PDB code ; 2aq8 (ref. 10)) and with INH–NAD (PDB code ; 1zid^5^); the S94A mutant in complex with NADH (PDB code ; 4dti^11^) and with INH–NAD (PDB code ; 2nv6 (ref. 7)). In the case of both structures with the INH–NAD inhibitor bound, the reported structures feature a mutation at residue 2 and not all were complete sequences in the protein databank with early residues missing. These variations have been accounted for in the residue numbers reported from calculations. It is noted that InhA exists as a homotetramer *in vivo*, but the crystal structures are for the monomeric unit only and these were studied in the simulations. Extensive simulations of the tetramer have revealed no correlated motions over 150 ns among the four pockets, supporting the assumption that their movements can be treated as independent.^29^

### MD simulations and IR calculations

MD simulations were carried out starting from each of the four crystal structures of InhA listed above. Simulations of the apo-form were modelled by excising the co-factor or inhibitor prior to performing simulations. The structures were minimised using the steepest descent algorithm and solvated in a truncated octahedron of TIP3P water,^30^ ensuring a minimum distance of 10 Å between the protein and the edge of the water box. A simulation was initially carried out with the protein atoms restrained for 50 ps in order to “soak” the peptide backbone in the water molecules. Subsequently, an equilibration period of 5 ns was carried out in an NVT ensemble at 300 K. Periodic boundary conditions and PME electrostatics^31^ were used with a cut off of 10 Å and the Verlet scheme^32^ to allow GPU acceleration of the simulation in GROMACS.^33^,^34^ Heavy atom-hydrogen bonds were constrained using the LINCS algorithm,^35^,^36^ permitting a 2 fs time step size. Production dynamics were carried out using the same parameters for 20 ns, with snapshots saved every 1 ps. The ligand simulations were set up with the CHARMM-GUI^37^ using the CHARMM36 forcefield.^38^

For simulations of the inhibited enzymes, parametrisation of INH–NAD was performed using the CHARMM Generalised Forcefield^39^ and the SwissParam server.^40^ For simulations of the enzyme with the co-factor bound (based on PDB: ; 2aq8 (ref. 10) and ; 4dti^11^), the *N*-terminal glycine was patched using the GLYP patch. All four structures were solvated as for the apo-enzyme simulations. The solvated structures underwent 30 000 steps of minimisation using the steepest descent and the Adopted Basis Newton–Raphson schemes. This was followed by a 10 000 step minimisation using the conjugate gradients scheme and a 50 ps dynamics run carried out to ensure no bad contacts between the enzyme and ligand. The production dynamics were performed using GROMACS in order to take advantage of the GPU acceleration. All other simulation details were the same as for the simulations of the apo-forms.

Twelve independent simulations were performed for each system. FTIR spectra in the amide I region were computed using an exciton approach.^41^,^42^ A one-quantum exciton Hamiltonian was constructed, with the off-diagonal elements representing interaction energies between local amide I vibrations computed using the transition dipole coupling model^43^ except for the nearest–neighbour interactions. The latter were taken from a look-up table of couplings derived from DFT calculations on a systematic set of conformations of a dipeptide.^44^ Network graphs^45^ generated using the open-source code gephi^46^ were used to visualize the inter–residue interactions.

A scaled two-quantum Hamiltonian, comprising two-quantum local states and coupling, was constructed^41^,^42^ from the one-quantum Hamiltonian matrix elements, using a modified version of the peptide C programme of Hamm and Zanni.^19^ This programme was used to compute the 2D signal using 250 snapshots from a single representative trajectory. In previous work,^42^ we found that for the small protein ubiquitin, where more extensive exploration of convergence was tractable, 2D-IR spectra computed with 200 uniformly sampled snapshots gave a computed spectrum very similar to that computed with 2000 snapshots. The non-zero two-quantum Hamilton matrix elements are:*H*_*ij*,*ij*_ = *H*_*i*,*i*_ + *H*_j,j_ – *δ*_*i*,*j*_*ΔH*_*ii*,*jk*_ = √2 (*H*_*i*,*j*_*δ*_*i*,*k*_ + *H*_*i*,*k*_*δ*_*i*,*j*_); *ii* ≠ *jk*, *j* ≠ *kH*_*ij*,*jk*_ = *H*_*i*,*k*_(*δ*_*i*,*j*_ + *δ*_*j*,*k*_); *i* ≠ *k*

The different sites (local amide I vibrations) are indexed by *i*, *j* and *k*; *δ* is the Kronecker delta. The anharmonicity, *Δ*, was taken to be 16 cm^–1^.^47^ The computed 2D signal is the sum of the rephasing and nonrephasing components. It is assumed that the transition dipoles scale like a harmonic oscillator from the fundamental to the overtone and that the dephasing times of the 0 → 1 and 1 → 2 transitions are equivalent.

## Results

For the wild-type enzyme and each of the mutations studied, activity assays were carried out both in the presence and absence of the INH–NAD inhibitor to establish the viability of the enzyme samples. This data has been reported elsewhere^22^ but, briefly, the results are consistent with previous studies of the wild-type and S94A mutation, showing an affinity for NADH in the μM range while the INH–NAD inhibitor binds with nM affinity.^2^,^3^,^9^ It has been shown that the S94A mutation results in a reduction in affinity for NADH (though this remains in the μM range) but that the mutation has little impact upon INH–NAD binding.^2^,^3^,^9^ In the case of the additional W222A mutation studied here, this showed a slightly reduced activity, with respect to NADH turnover, in relation to the wild-type enzyme (∼60%) and was found to be inhibited by INH–NAD.^22^ The P193A mutation was found to have negligible activity with respect to both NADH and INH–NAD binding and will therefore provide a negative control for the spectroscopy data.

To place the results in a structural context, the InhA structure features a Rossmann fold co-factor binding site consisting of two β-strands (designated str-4 and str-5) flanking the helix (hel-5) which contains the K165 and Y158 residues that have been shown to be key to InhA function (Fig. 1(c)).^2^,^9^,^48^ Two further α-helices (hel-4 and hel-6) complete the binding pocket.^48^,^49^ In studies of InhA with co-factor and substrate bound it has been shown that the residues 195–207, which contribute to hel-6 and form the substrate binding loop (SBL, Fig. 1(c)), can undergo a disorder–order transition that may influence substrate binding.^2^

### FTIR spectroscopy shows that no gross structural changes occur upon ligand binding, but the 1620 cm^–1^ part of the amide I band of wild-type InhA increases in amplitude

**Fig. 2:**
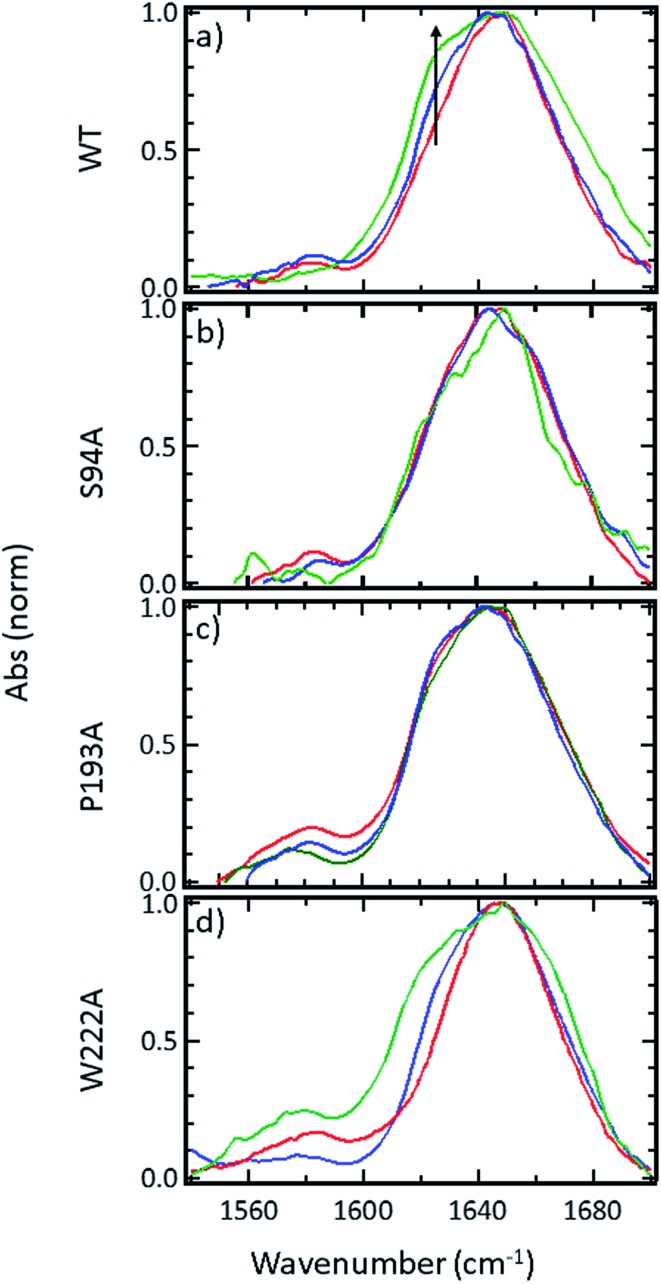
FTIR spectra of (a) wild-type InhA (b) S94A–InhA, (c) P193A–InhA and (d) W222A–InhA. In each panel red corresponds to the apo-enzyme, blue to the enzyme with NADH cofactor present and green to the enzyme with the INH–NAD inhibitor present. The arrow in (a) marks the increased amplitude near 1620–1630 cm^–1^ observed upon binding of the co-factor and inhibitor to the wild-type enzyme discussed in the text. All spectra are normalised to the peak of absorbance. Uncertainty due to measurement error in each individual spectrum is indicated by the width of the line.

A representative selection of the FT-IR spectra for the apo enzymes and enzymes with co-factor or inhibitor present are shown in Fig. 2. In light of the availability of crystal structures for the wild-type enzyme (Fig. 2(a)) and S94A mutation (Fig. 2(b)), we will focus on these in the discussion, using the P193A mutant (Fig. 2(c)) as a control. The data for the W222A mutant is shown in Fig. 2(d) for comparison. In these figures, the red trace represents the apo-enzyme; blue represents the enzyme with the NADH co-factor present and green the enzyme with the INH–NAD inhibitor present.

In the amide I region of the IR spectrum, a broad absorption band is observed in each case centred near 1640 cm^–1^ as would be expected for an enzyme with a secondary structure that is largely α-helical in composition.^15^–^19^ The similarity of the spectra shows that no major change in structure of the enzyme is occurring in any of the samples. The data in Fig. 2 has been normalised to the peak absorption in order to compare the relative shapes of the absorption bands. In the case of the wild-type enzyme (Fig. 2(a), red), a slight broadening of the amide I lineshape is observed upon binding of the NADH co-factor (Fig. 2(a), blue) and a shoulder is observed on the low-frequency side of the band located near 1620–1630 cm^–1^. The latter is shown by an arrow in Fig. 2(a) and becomes more pronounced when the INH–NAD inhibitor is bound to the enzyme (Fig. 2(a), green). In the case of the apo-forms of the S94A mutation and P193A control (Fig. 2(b) and (c), red) a slight asymmetry is observable on the low frequency side of the lineshape in the position of the 1620–1630 cm^–1^ feature observed in the wild-type enzyme spectra. This is slightly more pronounced in the P193A case (Fig. 2(c), red). However, the feature does not change significantly upon either co-factor or inhibitor binding in either mutation in relation to the signal to noise level achievable at the concentration studied. It is important to stress that, under the conditions described for these measurements, the size of the absorption due to the co-factor or inhibitor was negligible and that any changes observed are occurring to the protein as a result of binding. No aggregation of the protein was observed. The results for the W222A mutant most closely resemble those of the wild-type enzyme (Fig. 2(d)).

### 2D-IR spectroscopy reveals an off-diagonal peak with an amplitude that correlates with biological activity

**Fig. 3:**
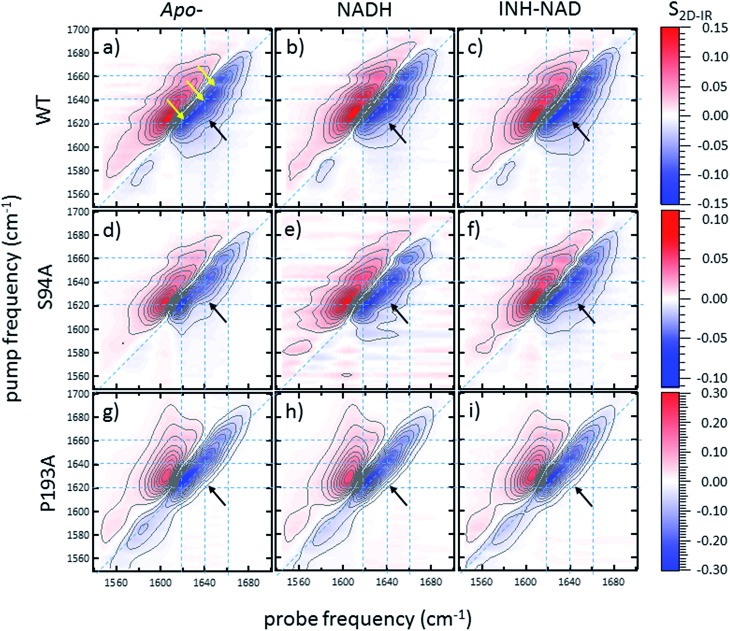
2D-IR spectra of (a–c) wild-type InhA (d–f) S94A–InhA and (g–i) P193A–InhA. In each case, spectra are shown for (a, d, g) the apo-enzyme, (b, e, h) the enzyme with NADH cofactor present and (c, f, i) the enzyme with the INH–NAD inhibitor present. Scale bars (*S*_2D-IR_) show the magnitude of the 2D-IR signal, a total of 20 contour lines are shown at evenly-spaced 5% intervals of the maximum signal shown. Yellow arrows in (a) mark the position of individual components of the *v* = 0–1 diagonal peak discussed in the text. The black arrows show the position of the off-diagonal feature discussed in the text.

Ultrafast 2D-IR spectra of apo–wild-type InhA and wild-type InhA with co-factor and inhibitor bound are shown in Fig. 3(a–c) along with the corresponding spectra for the S94A mutant (Fig. 3(d–f)) and P193A control (Fig. 3(g–i)). The spectra recovered are broadly similar in all cases, as would be expected for largely similar enzymes, consisting of a negative feature (blue) located on the spectrum diagonal with a positive feature (red) shifted to lower probe frequency by around 20 cm^–1^. Both components of the spectrum are strongly elongated along the diagonal. The negative feature has its centre near 1640 cm^–1^ on the spectrum diagonal and can be assigned to the *v* = 0–1 transition of the amide I mode of the enzyme, as observed in the FT-IR spectrum. The red feature is assigned to the amide I *v* = 1–2 transition; the shift of this feature along the probe axis arises from the anharmonicity of the potential surface and is consistent with the accepted value of ∼16 cm^–1^ for the amide I mode.^47^

It can be seen from Fig. 3(a) that the amide I *v* = 0–1 band is not a simple feature and that three individual components occur along the diagonal located near 1620, 1640 and 1660 cm^–1^ (see guidelines and yellow arrows in Fig. 3(a)). Fitting of the FT-IR spectra in Fig. 2 revealed that these components can also be seen in the absorption spectra (Fig. S2†) but they are better-resolved by the 2D-IR methodology, which accentuates narrow features due to the dependence of the 2D-IR signal upon the fourth power of the transition dipole moment.^47^ The features near 1640 cm^–1^ and 1620 cm^–1^ can be assigned, based on previous work on protein IR spectroscopy, to α-helix and β-sheet components of the secondary structure respectively. The 1660 cm^–1^ feature is attributable to β-turn components.

In the off-diagonal regions of the 2D-IR spectra in Fig. 3, the most prominent feature links the diagonal peaks near 1620 and 1640 cm^–1^. This is much more clearly resolved in cross-section (see below) but is marked by black arrows in Fig. 3 and was observed in each of the enzymes studied. Off-diagonal features in 2D-IR spectra generally indicate the presence of vibrational coupling, energy transfer or chemical exchange.^15^–^19^ Given the short waiting time at which the spectra were recorded (250 fs), the effects of energy transfer and exchange are assumed to be negligible and the likely assignment in this case is to vibrational coupling patterns associated with residues involved in the β-sheet element of the InhA structure.^50^

**Fig. 4:**
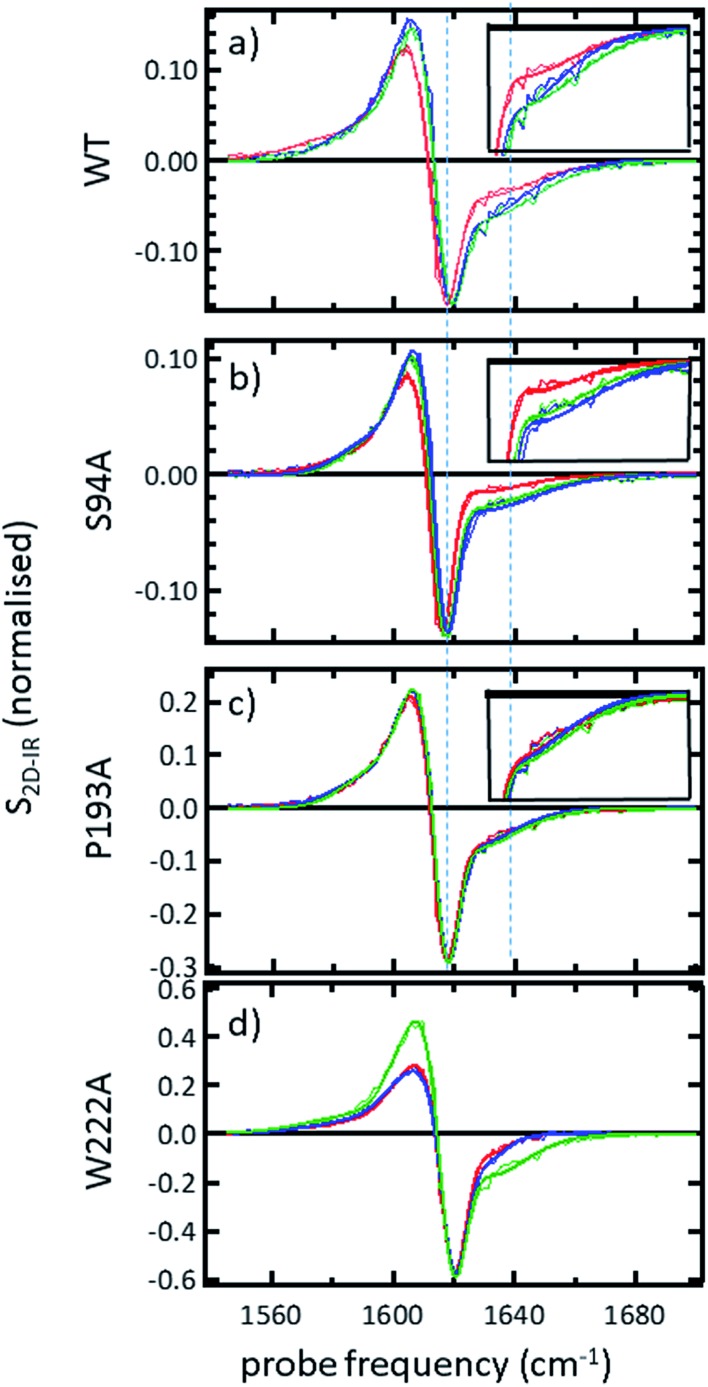
(a–c) Slices through 2D-IR spectra at a pump frequency of 1620 cm^–1^ of (a) wild-type InhA (b) S94A–InhA (c) P193A–InhA and (d) W222A–InhA. Red: apo-enzyme; blue: NADH cofactor bound; green: INH–NAD inhibitor bound. Insets in (a–c) show expanded regions near the off-diagonal feature discussed in the text. Uncertainty due to measurement error in each individual spectrum is indicated by the width of the line.

In order to more clearly differentiate between the off-diagonal features observed in Fig. 3, cross sections through the 2D-IR spectra were taken at a pump frequency of 1620 cm^–1^ for the wild type (Fig. 4(a)), S94A mutant (Fig. 4(b)), P193A control (Fig. 4(c)) and W222A (Fig. 4(d)) in the apo-enzyme (red), enzyme with co-factor (blue) and enzyme with inhibitor (green) forms. Here, the diagonal (*v* = 0–1) peak at 1620 cm^–1^ and the off-diagonal feature near 1640 cm^–1^ are resolved; vertical dashed lines in the figure mark the positions. In the case of the wild-type enzyme (Fig. 4(a)), when NADH is present (Fig. 4(a), blue), the off-diagonal feature grows in size in comparison to the apo-enzyme (Fig. 4(a), red). Addition of INH–NAD (Fig. 4(a), green) also increases the amplitude of the off-diagonal feature, but the peak also appears broadened and shifted slightly to higher wavenumber in comparison to that with NADH alone.

**Fig. 5:**
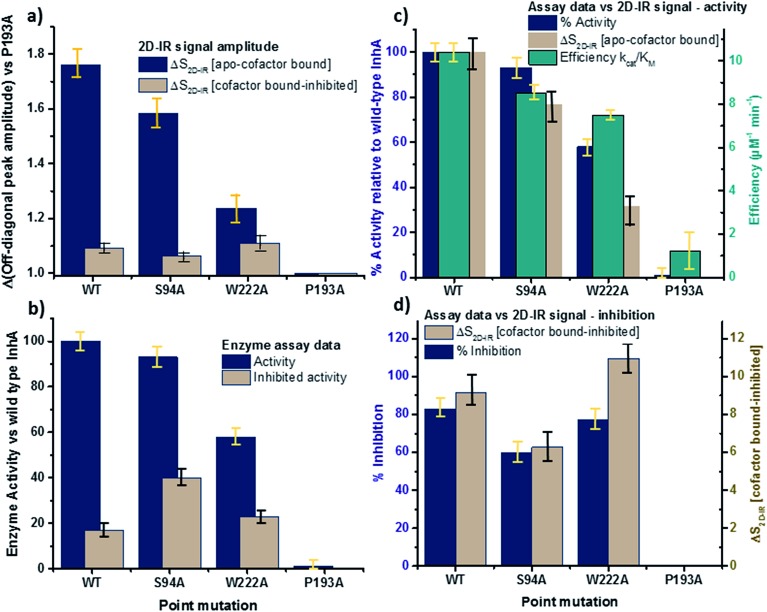
Comparisons of enzyme assay data with amplitude changes of the off-diagonal peak from Fig. 4. (a) Fractional change of the amplitude of the off-diagonal peak at [pump, probe = 1620 cm^–1^, 1640 cm^–1^] on binding co-factor (blue) and changing co-factor to inhibitor (brown) (b) enzyme assay activity results (blue) given as percentage of wild-type enzyme activity. Inhibition values (brown) show activity following inhibition as a percentage of the value for the uninhibited mutant.^22^ NB quoted S94A data from ref. 22 were obtained using higher INH–NAD concentration than wild type and W222A so inhibited activity represents a lower limit. (c) Compares the change in amplitude of the off-diagonal peak upon co-factor binding (brown) with enzyme activity (blue) and efficiency (green) derived from Michaelis–Menten analysis.^22^ (d) Directly compares the off-diagonal peak amplitude change upon changing co-factor to inhibitor (brown) with the level of inhibition blue, the inverse of the inhibited activity data from (b). NB quoted S94A data from ref. 22 were obtained using higher concentration of inhibitor than wild type and W222A so inhibition represents an upper limit. Error bars on 2D-IR data indicate the spread of sample-to-sample variation from triplicate measurements, those on biological data are taken from ref. 22.

The changes in the off-diagonal peak were quantified by fitting the 1620 cm^–1^ cross-section of the 2D-IR spectra to Gaussian lineshape functions. The results of the process are shown in detail in Table S1 and Fig. S3.† To account for variations between individual measurements arising from fluctuations in laser intensity or concentration, we focus on the ratio of the amplitudes of the off-diagonal to the diagonal peaks in this cross-section. Applying this analysis to the wild-type enzyme data shows that, upon binding of NADH, the off-diagonal feature increases in amplitude from 21% of the diagonal *v* = 0–1 feature to 37%; this increases further to 42% for the inhibited wild-type enzyme. The fitting results also show that the off-diagonal feature is centred near 1630 cm^–1^ and suggests that a shift to higher frequency and a broadening occurs upon binding the co-factor, with a further shift and broadening upon inhibition. However, the changes in peak position and width are of the order of 2–3 cm^–1^, comparable with the resolution of the instrument and so the main result is a change in amplitude of the feature upon binding of the co-factor followed by a further increase upon inhibition. The fractional increases in the ratio of the amplitude of the off-diagonal peak to the diagonal feature derived from lineshape fitting are displayed in Fig. 5(a) for binding of the co-factor (blue) and changing the co-factor to the inhibitor (brown). For the wild-type enzyme, the increase in off-diagonal peak amplitude ratio from 21% to 37% gives a fractional increase of 1.76 in Fig. 5(a), a further increase by a factor of 1.14 is indicated by the curve fitting analysis upon changing NADH to INH–NAD (37–42%).

When studying the amide I band of the whole enzyme molecule, changes to the 2D-IR signal caused by binding of a ligand that has been shown not to affect the structure of the molecule in a significant manner are likely to be subtle. It is thus important to put the results obtained for the wild-type enzyme into context by examining the cross sections through the 2D-IR spectra of the P193A control, which showed negligible activity or response to INH–NAD in enzyme assays.^22^ The same off-diagonal feature is present in the P193A apo-enzyme (Fig. 4(c)) but it does not change in terms of amplitude or position/width in response to addition of either NADH or INH–NAD (Fig. 4(c), Table S2†). Crucially, the three spectral slices overlap to within the noise level of the measurement, such that they are indistinguishable for the control sample (Fig. 4(c)). Using the same curve-fitting approach as applied to the wild type enzyme data shows that the ratio of the amplitude of the off-diagonal feature to that of the diagonal peak varies by less than 1% across the three samples (Table S2,† Fig. 5(a)). This gives us confidence in the validity of the subtle spectral changes observed upon inhibition of the wild-type enzyme and we use bars on the data in Fig. 5(a–d) to give an indication of the observed sample-to-sample variation in the results because, for protein samples, these variations far outweigh the measurement errors in a single sample. It is noted that the molecular impact of introducing INH to the system is the addition of a relatively small aromatic ring to the large NAD molecule and so a larger impact upon the enzyme spectrum would be anticipated to occur upon cofactor binding rather than during the change from cofactor to inhibitor binding, as we observe.

Extending the study to the S94A mutant (Fig. 4(b), red) shows that the apo-enzyme spectrum also contains the same off-diagonal feature, but it is considerably lower in amplitude than that of the wild-type apo-enzyme (12% of the diagonal *v* = 0–1 feature, Table S3†)). Upon binding of NADH, this feature again increases in size to 19% of the *v* = 0–1 peak amplitude (Fig. 4(b), blue and Fig. 5(a)). In the case of the S94A mutant, binding of the inhibitor complex results in similar value for the off-diagonal peak amplitude as observed for the co-factor; the curve fitting analysis indicates a very small amplitude increase to 21% upon inhibition (Fig. 5(a)). Once again a slight broadening and shift to higher frequency of 2–3 cm^–1^ is also indicated. Although the changes in the off-diagonal peak are more subtle than observed for the wild-type enzyme, they are larger than the variation between spectra observed for the P193A control.

In the case of the W222A mutation (Fig. 4(d)), which behaved in a similar manner to the wild-type enzyme in assays, though with reduced activity, a similar trend is observed in the 2D-IR spectra in this mutant to the wild-type enzyme. The off-diagonal feature increases in amplitude from 21 to 26% and 30% of the *v* = 0–1 diagonal feature upon binding of the co-factor and inhibitor respectively (Table S4,† Fig. 5(a)).

## Discussion

The off-diagonal feature linking the parts of the diagonal lineshape located at 1620 and 1630–40 cm^–1^ in the 2D-IR spectrum is responsive to point mutation and co-factor or inhibitor binding. It is widely accepted that very little macroscopic change in the structure accompanies these processes and so the 2D-IR data is potentially indicative of a new molecular response to ligand binding.^5^,^7^,^10^,^11^

We evaluate the significance of this result by comparing the spectroscopic data to the results of biochemical assays of enzyme performance, beginning by considering the change in amplitude of the off-diagonal feature upon NADH binding. It is established that point mutations can influence the affinity of InhA for NADH and the activity of each mutant relative to the wild-type enzyme is shown in Fig. 5(b) (blue bars).^22^ In Fig. 5(c) the change in the off-diagonal peak amplitude upon co-factor binding is compared to enzyme activity and another measure of enzyme performance, the efficiency, determined as *k*_cat_/*K*_M_*via* a standard Michaelis–Menten analysis.^22^ The striking result is that the amplitude change of the off-diagonal peak in the spectrum of each mutant upon co-factor addition correlates well with measurements of enzyme activity Fig. 5(c). The level of variation for W222A is greater than the wild-type or the S94A mutant, but this is also true of the assay data, which shows greater variance between activity (determined from initial reaction rates) and efficiency parameters, suggesting a more complex biochemical mechanism may be operational in this mutant. Overall, the correlation of the amplitude of the off-diagonal feature with co-factor binding is strongly indicative of a relationship between spectroscopy and enzyme function.^22^

In the case of all mutants studied except the control, a further, smaller, increase of the off-diagonal peak was observed upon changing the co-factor (NADH) to the inhibitor (INH–NAD). Comparing spectroscopic and biochemical assay data suggests that the change in the amplitude of the off-diagonal feature upon moving from co-factor binding to inhibition closely follows the impact of the inhibitor upon enzyme function. Fig. 5(a) and (b) show that the largest change in the shoulder amplitude upon inhibition occurs for the INH-susceptible wild-type and W222A mutants, whereas that for S94A is smaller (Fig. 5(a)), consistent with the reduced inhibition experienced by this resistance-implicated mutation (Fig. 5(b)). The correlation is shown by Fig. 5(d), which compares the level of inhibition of each enzyme with the change in the off-diagonal peak amplitude, where the two trends follow each other very closely. This demonstrates clearly that inhibition has an additional molecular impact over and above co-factor binding that is detected by 2D-IR spectroscopy. That the region of the spectrum affected is the same in both co-factor and inhibitor binding implies that the molecular origin may be similar, perhaps as would be expected given that the same binding site is accessed by both co-factor and inhibitor, but, crucially, the extent of the spectroscopic effect differs.

Before turning to the modelling aspects of this study it is important to consider the possible origins of the off-diagonal component that we observe in the 2D-IR spectra of InhA. As described above, it is clear that the feature is assignable to vibrational coupling between an excited mode, with a transition frequency of 1620 cm^–1^, and others with a transition frequency of 1630–1640 cm^–1^. The 1620 cm^–1^ mode is assigned to the *ν*_perpendicular_ mode of a β-sheet secondary structure element in which oscillators that line up across the strands couple strongly through hydrogen bonding.^50^ The nature of the modes at 1630–1640 cm^–1^ are less clear however and we discuss three possibilities as to the assignment of these modes. The first possibility is that the features are due to coupling patterns within the β-sheet. Although the *ν*_perpendicular_ mode is prominent in a β-sheet spectrum, many other modes contribute and some of these have frequencies in the 1630–1640 cm^–1^ region of the spectrum.^19^ In an infinite, well-ordered anti-parallel β-sheet these are forbidden by symmetry and a second *ν*_parallel_ mode is observed near 1680 cm^–1^. In reality, these weaker modes are visible leading to a continuum feature, arising from an assortment of low intensity transitions, being observed between the strong 1620 and 1680 cm^–1^ modes. The 2D-IR spectrum of an antiparallel β-sheet has been reported to show coupling patterns linking the main *ν*_perpendicular_ and *ν*_parallel_ transitions to weaker modes located near 1630–1640 cm^–1^, giving the 2D-IR spectrum a characteristic ‘*Z*’-shape signature.^50^

The InhA co-factor binding site features a parallel β-sheet and this leads to a change in the mode structure expected. The *ν*_perpendicular_ mode is still present but the 1680 cm^–1^*ν*_parallel_ transition is not observed and is predicted to occur at a much lower frequency, below 1640 cm^–1^, in the parallel case.^51^ A similar raft of low intensity modes are also expected to be visible in a non-perfect parallel β-sheet and so the off-diagonal feature shown in Fig. 3 is consistent with changes in couplings involving the *ν*_perpendicular_ mode of the parallel β-sheet of the Rossmann fold. A better-defined β-sheet arising from binding could increase the size of the β-sheet signal and potentially increase the visibility of the off-diagonal component linking to the *ν*_parallel_ mode at frequencies below 1640 cm^–1^. The localised position and relatively narrow width of the off-diagonal feature indicated by fitting of slices through the 2D-IR spectrum along with the increased 1620 cm^–1^ amplitude in the IR absorption data (Fig. 2(a)) would seem to support assignment of this to a well-defined vibrational mode.

The second possibility is that an increase in disorder within the β-sheet upon co-factor binding leads to the weaker modes gaining prominence relative to the diagonal peak by relaxing the symmetry-forbidden nature of the transitions. The localised and narrow nature of the off-diagonal peaks observed and the increase in the β-sheet signal at 1620 cm^–1^ observed in the absorption spectra tends to favour the increased integrity argument over this explanation however. The fact that a significant increase in the enzyme melting temperature has been reported upon binding of both NADH and INH–NAD also contradicts this argument.^12^ This was interpreted as an increased stabilisation of the structure and would be consistent with a general increase in coupling interactions within the molecule rather than increased disorder.

The third possibility that may cause altered vibrational coupling patterns of the *ν*_perpendicular_ β-sheet mode is changes in coupling of β-sheet modes to α-helix modes, which typically occur near 1635 cm^–1^. As the central feature of the InhA co-factor binding pocket is the β–α–β Rossmann fold, this assignment would seem reasonable, though the fitting results suggest that the feature may occur at too low a frequency to be purely due to β–α coupling.^47^

Overall, the data are consistent with a picture in which binding of the co-factor to all but the inactive P193A mutant leads to an enhanced β-sheet spectral contribution, potentially with increased off-diagonal components arising from the β-sheet itself or interactions between the β-sheet and α-helix elements of the Rossmann fold. Further changes are caused by inhibition.

### Molecular modelling and spectra calculations assign off-diagonal peak to changes in vibrational coupling that permeate the InhA structure

We now refer to modelling studies to shed more light on these scenarios, but it is clear from experiment that the β-sheet is central to the spectral changes and is altered by binding. This is consistent with its prominent place in the co-factor binding site of InhA. It is however noted that, given the structural complexity of the co-factor binding pocket, multiple mechanisms may contribute, for example increased β-sheet–α-helix coupling accompanying an enhanced β-sheet signal.

**Fig. 6:**
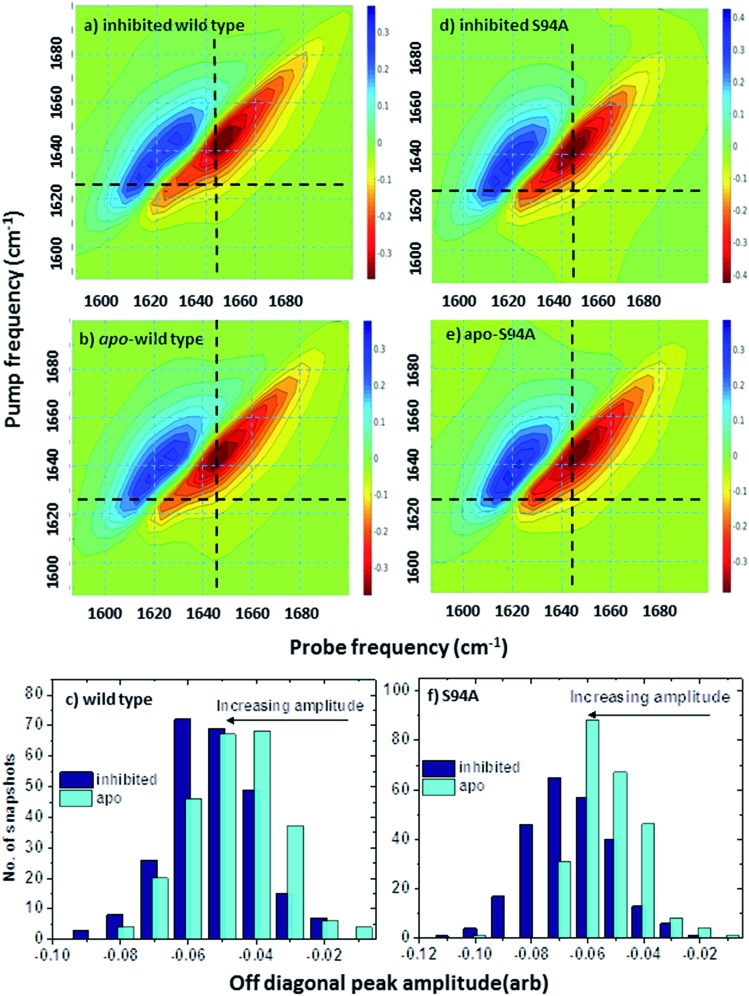
Simulated 2D-IR spectra of InhA. (a) Simulated spectrum based on the structure of the wild type enzyme with the inhibitor complex bound (PDB: 1zid^5^) (b) simulated 2D-IR spectrum showing the effect of excising the inhibitor ligand from the structure used to produce the spectrum in (a). (c) Bar graph showing distribution of off-diagonal peak amplitudes for inhibited (dark blue) and apo-forms of the wild-type enzyme calculated from 250 individual simulation snapshots. The shift to larger (negative) predicted peak amplitudes upon inhibition is clear. (d) Simulated spectrum based on the structure of the S94A mutant with the inhibitor complex bound (PDB: ; 2nv6 (ref. 7)) (e) simulated 2D-IR spectrum showing the effect of excising the inhibitor ligand from the structure used to produce the spectrum in (c). Black dashed lines mark the position of the off-diagonal feature discussed in the text. (f) Bar graph showing distribution of off-diagonal peak amplitudes for inhibited (dark blue) and apo-forms of the S94A mutant calculated from 250 individual simulation snapshots.

It is important first to establish that the proposed assignment of the spectroscopic signatures to changes in inter–residue couplings within the structure of the enzyme is reasonable. For this, atomistic simulations were used to calculate one-exciton Hamiltonian matrices and coupling interactions following MD simulations to reach an equilibrated structure of the ligand bound and ligand-free complexes. These were then used as the basis to calculate a two-quantum Hamiltonian and simulated 2D-IR spectra, examples of which are shown in Fig. 6. The simulated spectra clearly reproduce the main features of the experimental data in terms of the location of the *v* = 0–1 and *v* = 1–2 components of the signal. The general shapes of the 2D features match well with the experimental data (Fig. 3). Importantly, the presence of the off-diagonal component linking a pump frequency of ∼1620 cm^–1^ with a probe frequency of ∼1640 cm^–1^, as observed in the experimental data, is also present (marked by the intersection of the black dashed lines in Fig. 6) suggesting that the simulations are also reproducing the finer structure of the 2D-IR signal well. Comparison of the simulated spectra for the wild-type enzyme with inhibitor bound (Fig. 6(a)) and the wild type enzyme with the inhibitor excised (Fig. 6(b)) shows that the off-diagonal peak is smaller in magnitude the latter case (indicated by a lighter orange colour at the intersection point of the black guidelines), as was observed in the experimental data. This is displayed more clearly by the bar graph in Fig. 6(c), which shows the distribution of off-diagonal peak amplitudes predicted by 250 individual simulation snapshots. The shift of the graph to larger (negative) values upon binding is clear.

In the simulations of the corresponding spectra for the S94A mutant, the behaviour of the off-diagonal peak was also in good agreement with experiment, showing an increase in amplitude upon moving from the apo to the inhibited enzyme. The simulated spectra are shown in Fig. 6(d) and (e), the distribution of amplitudes from the simulation snapshots is shown in Fig. 6(f).

Contributions to the simulated 2D-IR spectra from the wild type and S94A mutation can be amplified further by exploiting the different responses of the diagonal and off-diagonal 2D-IR signals to polarisation in order to enhance the off-diagonal peaks.^19^ The result of plotting the simulated ZZZZ-ZXXZ signal, which has been shown to suppress diagonal peaks, is shown in Fig. S4† where the off-diagonal peaks appearing between the 1620 and 1640 cm^–1^ are clear and provide further evidence of the contribution from coupling of *β* secondary structural elements in the region of the spectrum observed in experiments.^42^

**Fig. 7:**
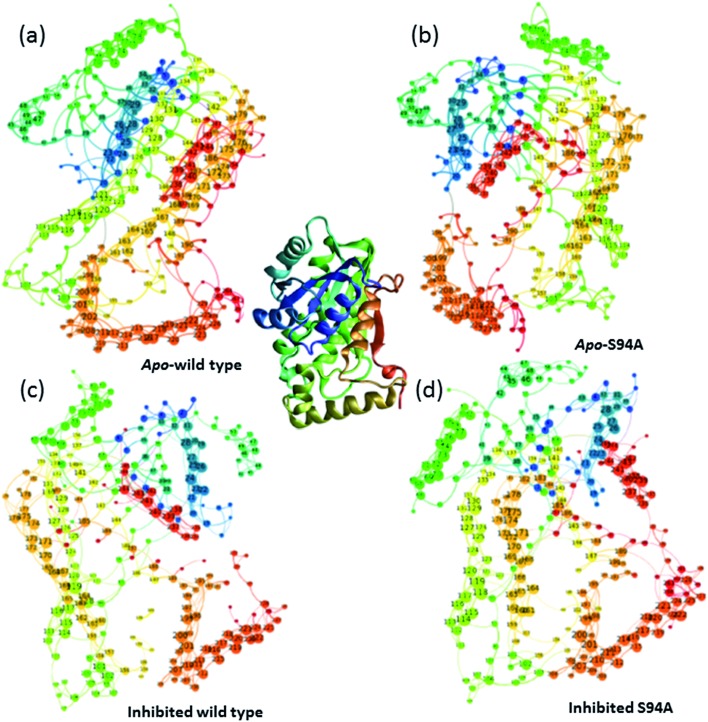
Network graphs depicting the one-quantum exciton Hamiltonian inter–residue couplings. (a) apo wild-type without inhibitor (PDB: 2aq8 (ref. 10)); (b) apo S94A mutant without inhibitor (PDB: ; 4dti^11^); (c) wild-type with inhibitor (PDB: ; 1zid^5^); (d) S94A mutant with inhibitor (PDB: ; 2nv6 (ref. 7)). The crystal structure (PDB: ; 1zid^5^) is shown as a ribbon diagram in the centre for comparison; the colours of residues are consistent across the figure but small changes in orientation of the network maps are present. Each node represents a residue; lines linking residues indicate a coupling interaction and the thickness of the line indicates the magnitude of the coupling, for further details see text.

Having established that the simulations reproduce the experimental data, it is instructive to explore the outcomes of the simulations in more detail. The results of calculating inter–residue couplings are displayed in Fig. 7 as network coupling maps^46^ where each node represents a residue; lines linking residues indicate a coupling interaction and the thickness of the line indicates the magnitude of the coupling. A comparison of the network maps produced for the enzymes using this approach shows excellent correlation with the crystal structures and the overall similarities between the PDB structures and the network model indicates that the parameters used in the models are of sufficient quality to draw conclusions about the structure and dynamics of the protein from the models. Elements due to the coupling patterns associated with residues participating in the α-helices and β-sheet are visible and it is clear that the former are dominated by near-neighbour couplings while the latter feature more couplings between strands in the sheet as would be expected.

**Fig. 8:**
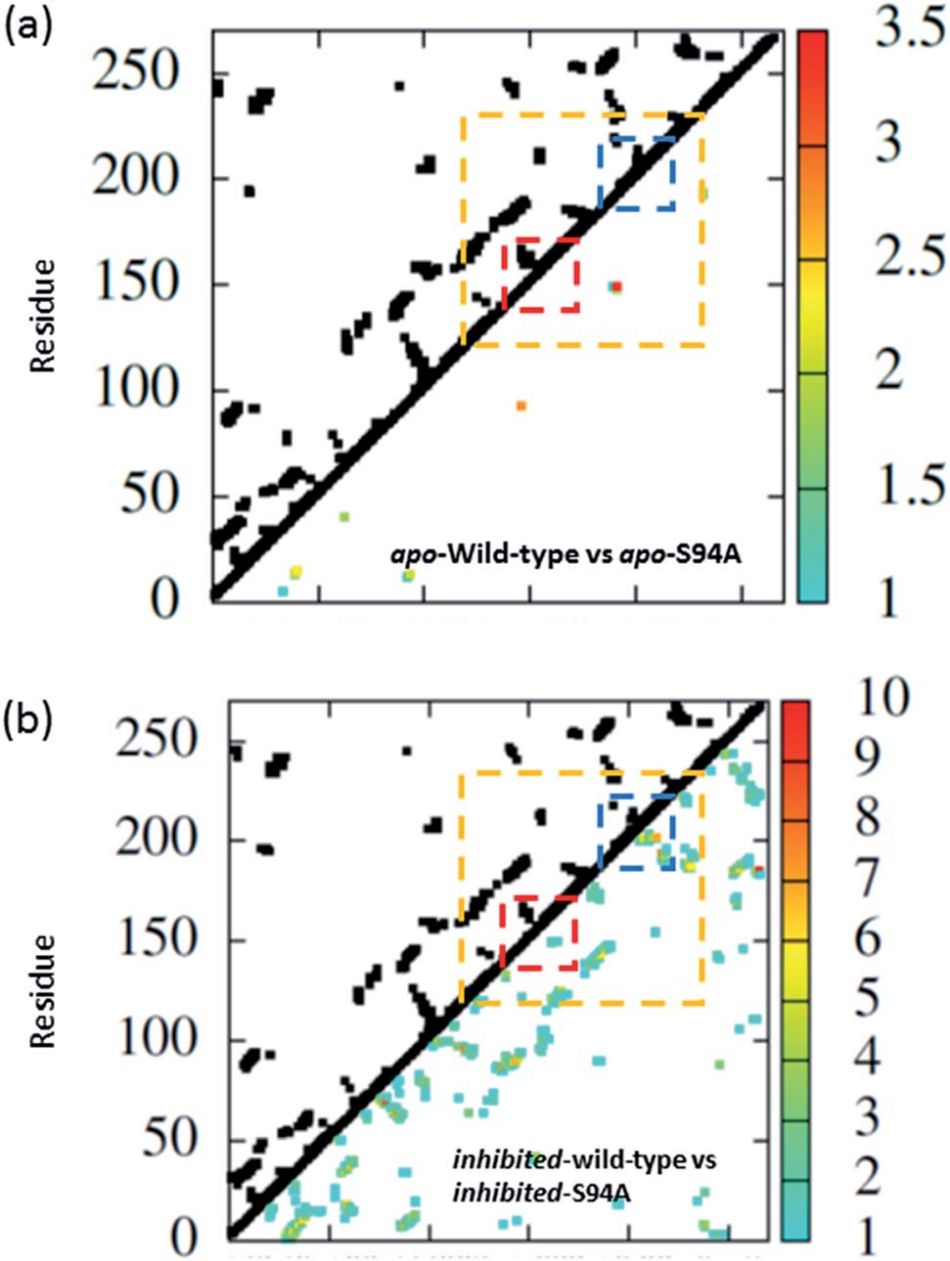
Coupling difference map (lower right quadrant) and contact map (upper left quadrant) for (a) apo wild-type without inhibitor (PDB: 2aq8 (ref. 10)) and apo S94A mutant without inhibitor (PDB: ; 4dti^11^); and (b) wild-type with inhibitor (PDB: ; 1zid^5^) and S94A mutant with inhibitor (PDB: ; 2nv6 (ref. 7)). A structural contact between two residues is depicted on the upper left quadrant of the map as a black square when the Cα atoms of the residues are within 10 Å of each other. Only interactions longer-range than *i*, *i* + 4 are shown. The lower right quadrant shows changes in vibrational coupling. Colour-bar scales depict the absolute difference in the coupling interaction energy (cm^–1^). Key regions of the structure are marked with boxes: orange: cofactor binding site; red: NADH interactions; blue: substrate binding loop.

The coupling networks are broadly similar for both apo-wild-type enzyme (Fig. 7(a)) and the apo–S94A mutant (Fig. 7(b)), indicating that the two are fundamentally similar in the absence of the binding ligand (either co-factor or inhibitor), consistent with observations from FT-IR data (Fig. 2) that the mutations do not cause large disruptions in the overall structure of the protein. A difference interaction map (Fig. 8) shows this more quantitatively than the network schematic. In Fig. 8, points along the diagonal represent amino acid residues. Black points above the diagonal, linking two residues, indicate spatial proximity of those residues while the region below the diagonal shows changes in couplings between the two structures that are compared. The change in coupling is shown *via* the colour-scale. Only couplings between residues separated by more than four positions in the amino acid sequence (*i*, *i* + 4) are shown in order to emphasise couplings within or between secondary structure elements. Key regions of the InhA structure are highlighted by coloured boxes; orange shows range of residues encompassing the cofactor binding site; red indicates residues proximal to the NADH cofactor; blue shows the substrate binding loop. It is clear from Fig. 8(a) that few significant differences in couplings are noted between the apo forms of the wild type enzyme and S94A mutant, as shown by the small number of points below the diagonal of the plot.

Introduction of the co-factor or INH–NAD inhibitor into each of the enzyme structures does not significantly change the structure observed (Fig. 7(c) and (d)), but the couplings are affected by inclusion of the ligand (Fig. 8(b)). In particular, the average coupling strengths are seen to increase in the inhibited enzyme and strong couplings are observed between residues in the Rossmann fold binding site with either co-factor or inhibitor bound. Importantly, given our results, greater variation in the coupling interactions between the wild-type and the S94A mutant were observed upon inhibitor binding; the impact of inhibition being reduced in the S94A mutant in comparison to the wild-type enzyme. Fig. 8(b) shows the difference map comparing the couplings in the inhibited wild type enzyme and S94A mutant and it is clear that, not only does the number of coupling changes increase in the wild-type enzyme, but that they permeate across much of the molecule as well as directly influencing the co-factor binding region and substrate binding loop. This suggests that the inhibitor has an effect on the wild type enzyme that is not replicated in the S94A mutant.

Overall, the one-exciton Hamiltonian calculations show a general “relaxation” of the 3D structure for all structures studied (wild type and S94A, each with NADH co-factor and INH–NAD inhibitor bound) upon removal of the ligand. Smaller coupling values are observed in the apo form and there are fewer strong interactions between residues in the co-factor binding site in simulations of the apo-enzyme, suggesting these residues sit further apart than when the ligand is present. When a ligand is bound, larger differences in the shorter range interactions (*i*, *i* + 4 or less) occur and larger couplings are observed in general, supporting the idea of generally stronger inter-residue interactions and correlate with the higher experimental melting temperature observed in the ligand-bound form.

These results from the modelling are again consistent with changes in the spectrum observed upon ligand binding and imply that the ligand leads to a modification in the interactions between residues, as would be anticipated upon ‘locking’ the enzyme structure into a particular conformation through intermolecular interactions. It is also important that INH–NAD binding does not have a uniform impact on the wild-type and S94A mutant, the effect being overall larger in the wild-type enzyme than the S94A mutant, again consistent with the experimental observations of an increased off-diagonal peak amplitude in the wild-type enzyme.

In an effort to shed light on the dynamic impact of co-factor or inhibitor binding, MD simulations were carried out beginning with the published crystal structures of the wild type enzyme complexed with NADH (PDB 2aq8 (ref. 10)) and with INH–NAD (PDB ; 1zid^5^). Simulations were performed on the complete complex structure and on the apo structure, which began from the same point but with the ligand excised from the binding site. The process was repeated for the same two complexes of the S94A mutant (PDB with NADH: ; 4dti;^11^ with INH–NAD: ; 2nv6 (ref. 7)).

**Fig. 9:**
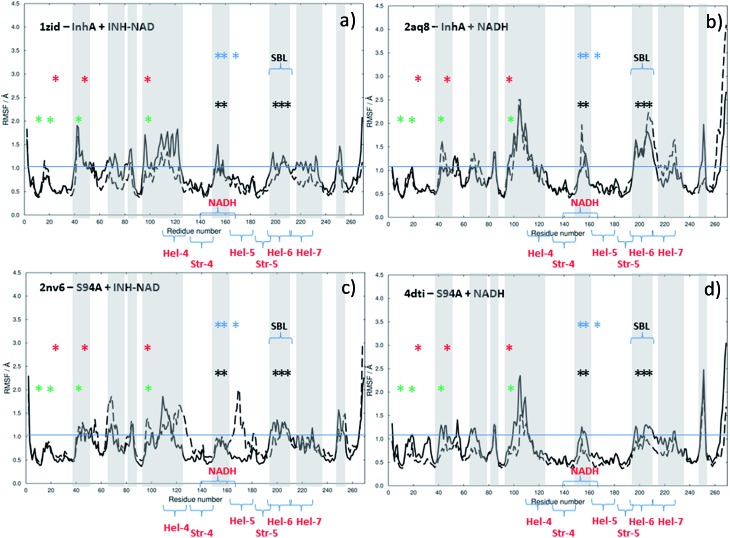
Results of MD simulations showing RMSF values as a function of residue number using crystal structures as starting points: (a) wild type InhA bound to INH–NAD, (b) wild type InhA bound to NADH, (c) S94A–InhA bound to INH–NAD, (d) S94A–InhA bound to NADH. In each panel, the solid line represents the apo enzyme, with the substrate removed, the dashed line represents the enzyme with co-factor or inhibitor bound. The horizontal lines are to guide the eye.

The results of this process are shown in Fig. 9. Each panel shows a comparison of the root mean square fluctuation (RMSF) of each residue of a particular enzyme mutant/ligand combination under apo (black solid line) and bound (dashed line) conditions. The grey areas are marked to show regions of interest and are identical across the four panels.

Notable residues obtained from biochemical or structural studies are marked using asterisks. Specifically, residues located near the INH part of the INH–NAD complex (Y158, F149, L207, I202, M199) are marked with black asterisks, while those affected by binding of NADH (16, 20, 41–44 and 95) are marked green. The Y158, F149 and K165 residues that have been shown to be key to function are marked with blue asterisks; Y158 and F149 have both been shown to move when the adduct binds with Y158 rotating towards the substrate binding loop. The latter (marked SBL in Fig. 9) is the region of hel-6 near residue 200 that has been observed to undergo an order-disorder transition. F149 has been observed to rotate to form a stacking interaction with the INH ring of the adduct. The mutations that have been observed to confer resistance in clinical isolates (S94A, I21V and I47T) are marked with red asterisks. The co-factor binding site is formed by str-4, str-5 and hel-5 plus hel-6 and hel-7, which are also marked.

Upon binding the INH–NAD inhibitor to wild type InhA (Fig. 9(a) solid line to dashed line), there is a general reduction in RMSF across the residue sequence. This is more notable in some regions of the structure than others but reductions in spatial fluctuations are predicted to occur near several of the important residues involved in either co-factor binding (black and green asterisks) or the portions of the InhA structure involved in function (blue asterisks and SBL). Hel-4, str-5 and hel-6, which contribute to the Rossmann fold, also experience reduced fluctuation upon ligand binding.

Comparing Fig. 9(a), for the inhibited wild-type complex to Fig. 9(b), which compares the results of MD simulations of RMSF for the wild-type-NADH complex with the apo-enzyme shows several differences. In particular, the general reduction in RMSF is not observed and the results for the ligand bound and ligand free complex are rather similar except for a surface-exposed residue, A154, in the NADH binding site and a portion of hel-6, which appear to experience more fluctuation.

Fig. 9(c) and (d) show the results for MD simulations of the INH–NAD and NADH complexes of the S94A mutation of InhA respectively. Here it is interesting to note that, in the case of the S94A–INH–NAD complex (Fig. 9(c)), the binding of the ligand has a less dramatic effect than in the case of the corresponding wild-type complex but appears to increase the RMSF rather than decrease it as in the wild-type enzyme. This is particularly notable in the NADH binding region near hel-4 and hel-5, though the SBL is unaffected. Similar to the wild-type enzyme, NADH binding to the S94A mutant seems to have a relatively modest effect on the predicted RMSF values.

In summary, MD simulations support the hypothesis that binding of the inhibitor to the wild-type enzyme has considerably more impact upon the dynamics of the key structural areas of the enzyme than NADH binding, leading to a general loss of flexibility of the structure. In the case of the resistant mutation S94A, this impact is apparently lost.

Overall, the combination of one-exciton Hamiltonian calculations and MD simulations suggest that the spectral changes observed in the 2D-IR data are linked to alterations in both the vibrational interactions and dynamics of the enzyme upon binding of co-factor and inhibitor. Binding of the co-factor leads to an increased vibrational coupling of the Rossmann fold residues in most cases, but binding the inhibitor leads to a dynamic restriction of the wild-type structure that is not seen in the S94A mutation.

## Conclusions

Bringing together the experimental and computation simulation results permits general conclusions to be drawn. The overall indication is that the increased amplitude of the off-diagonal feature observed in the 2D-IR spectroscopy upon ligand binding is likely to be attributable to a general increase in vibrational coupling occurring in the β–α–β Rossmann fold caused by binding of the co-factor and that this enhances the structural integrity of the β-sheet element of the structure, which is observed in the 2D-IR spectra. We suggest the additional increase in amplitude observed in the INH–NAD complex of the wild-type enzyme and W222A mutant that correlates with inhibition is due to reductions in overall flexibility of the enzyme in the inhibited case further increasing these effects. Although site-specific information is not obtainable from the 2D-IR dataset, the impact of occupation of the co-factor binding site upon both coupling changes and flexibility in the S94A mutant is markedly reduced, consistent with its function being less impaired by binding of the INH–NAD inhibitor.

The implications for enzyme action are interesting because the indication is that inhibition of wild-type InhA by INH–NAD is caused by the ligand ‘locking’ the structure in such a manner as to prevent function. This combines with the observation that changes in coupling and flexibility were not localised entirely to the binding site but spread throughout the structure (Fig. 8 and 9). This agrees with observations that the function of InhA may hinge upon its ability to form homo-tetramers or intermolecular complexes with other enzymes in the FASII pathway.^3^,^13^,^14^ In the inhibited wild-type case, the dynamic restriction imposed may prevent conformational searching or the ability to form a particular structure needed for this interaction. The more flexible S94A mutation can apparently circumvent this effect.

This study demonstrates that 2D-IR is able to reveal the subtleties of how binding of ligands to proteins leads to prolific changes to the protein scaffold away from the intermolecular contact site. These extended influences are expected to have important consequences for the overall structure and dynamics that lead to securing contact. We hypothesise that they promote drug efficacy and a sealing off of activity of the enzyme, with expected synergic effects. This work clearly shows a way forward to prove this hypothesis and characterise these synergic interactions by combining 2D-IR spectroscopy and MD simulations to address the problem of drug resistance in a systematic manner, providing a new means to identify potential drug targets.

## Conflicts of interest

There are no conflicts to declare.

## Supplementary Material

Supplementary information
