# Supplementary material for: Examining the role of protein structural dynamics in drug resistance in Mycobacterium tuberculosis
Source: Chem Sci. 2017 Oct 16;8(12):8384–99. doi: 10.1039/c7sc03336b (PMC5863454; doi:10.1039/c7sc03336b)
Supplement: Supplementary file 1 [file SC-008-C7SC03336B-s001.pdf]

Electronic Supplementary Material (ESI) for Chemical Science.
This journal is © The Royal Society of Chemistry 2017

Examining the role of protein structural dynamics in drug resistance in *Mycobacterium tuberculosis*

Daniel J. Shaw, [1†] Rachel E. Hill,[2] Niall Simpson,[1] Fouad S. Husseini, [2‡] Kirsty Robb,[3] Gregory M. Greetham,[4] Michael Towrie, [4] Anthony W. Parker, [4] David Robinson,[5] Jonathan D. Hirst,*[2] Paul A. Hoskisson*[3] and Neil T. Hunt*[1]

1) Department of Physics, University of Strathclyde, SUPA, 107 Rottenrow East, Glasgow, G4 0NG, UK

2) School of Chemistry, University of Nottingham, Nottingham, UK

3) Strathclyde Institute of Pharmacy and Biomedical Science, University of Strathclyde, Glasgow, UK

4) STFC Central Laser Facility, Research Complex at Harwell, Rutherford Appleton Laboratory, Harwell Science and Innovation Campus, Didcot, Oxon, UK

5) Department of Chemistry and Forensics, Nottingham Trent University, Clifton Lane, Nottingham, NG11 8NS, UK

† Present address: UCB Pharma, Slough, SL1 3WE, United Kingdom

‡ Present address: Department of Chemistry, University College London, Gower Street, London WC1E 6BT

** Corresponding author: neil.hunt@strath.ac.uk*

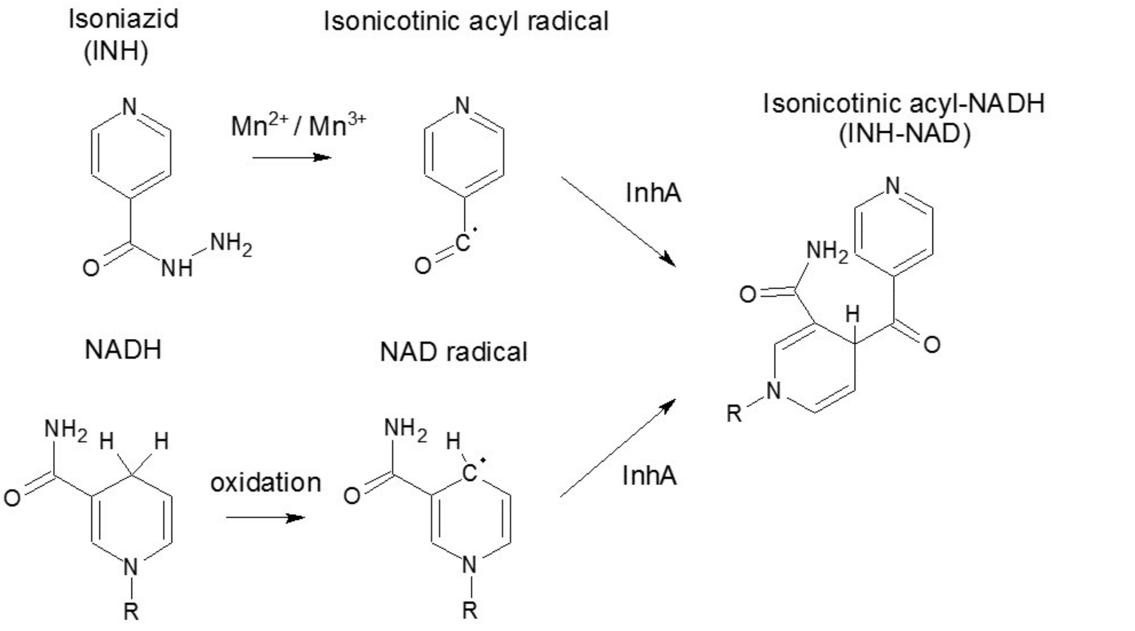

Scheme 1: *Proposed pathway for the formation of the INH-NAD adduct inhibitor of the enoyl reductase InhA (after main text Ref. 5). In vivo formation of isonicotinic acyl radicals is thought to be facilitated by* Mn^{2+}*. Also, the increased rate of* Mn^{2+} *to* Mn^{3+} *conversion by the native mycobacterial catalase-peroxidase KatG is thought accelerate radical formation.*

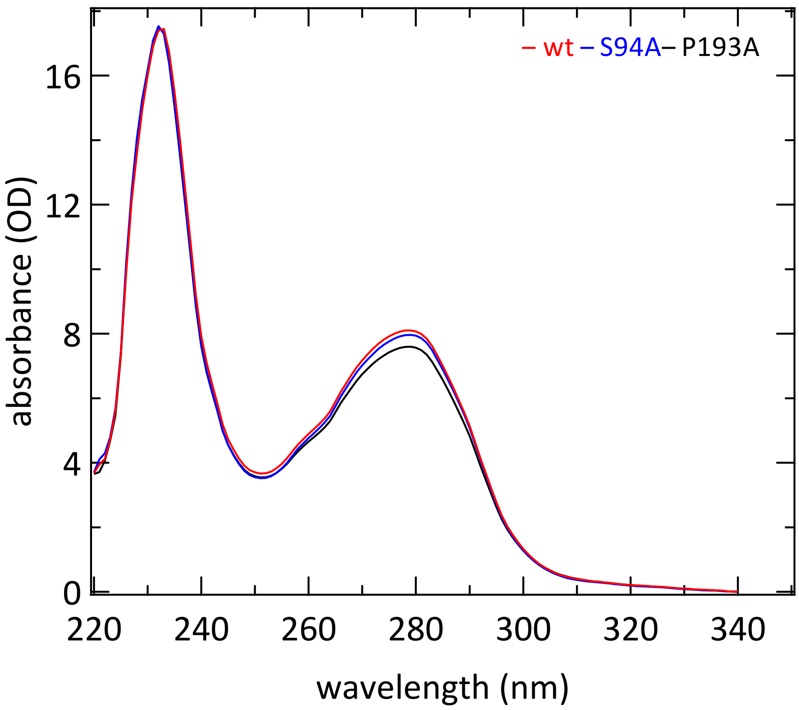

Fig.S1: Representative UV-vis absorption spectra of InhA mutants. Uncertainty due to measurement error in each individual spectrum is indicated by the width of the line.

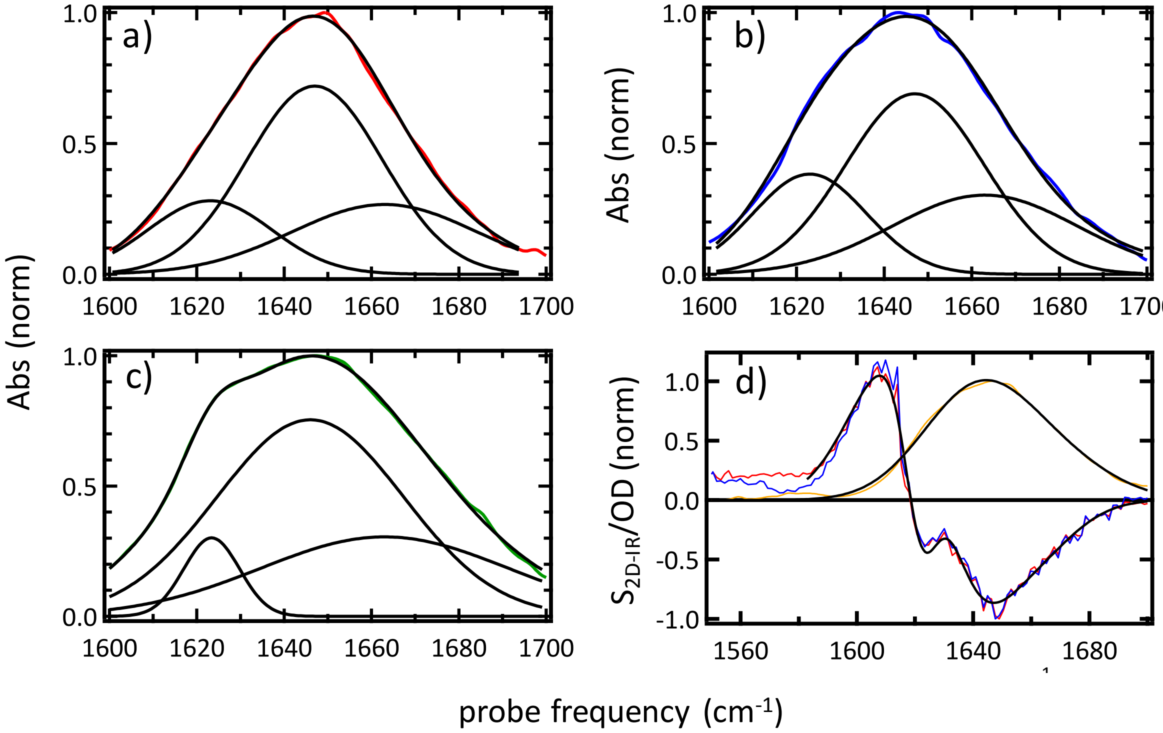

Fig S2: a)-c) FT-IR absorption spectra of wild-type InhA (solid black lines) along with results of fitting to a triple-Gaussian lineshape function (solid coloured lines) for a) apo-InhA, wild type b) wild type InhA with the NADH co-factor present and c) wild type with the inhibitor INH-NAD complex present. In each case the dashed lines show the individual contributions of the three lineshapes with central frequencies of 1623, 1647 and 1663 cm^{-1}. d) Shows a comparison of the FTIR spectrum (red) and diagonal of the 2D-IR spectrum of wild-type apo-InhA (blue). Black lines show the results of fitting the data to three Gaussian lineshapes centred at 1623, 1647 and 1663 cm^{-1}. The correspondence between the FTIR and 2D-IR diagonal lineshapes is clear, including the emphasis of the narrow 1623 cm^{-1} component in comparison to the broader features by 2D-IR spectroscopy as expected (see main text). The positive feature in the diagonal of the 2D-IR spectrum arises from the lineshape of the v=1-2 feature at low probe frequencies.

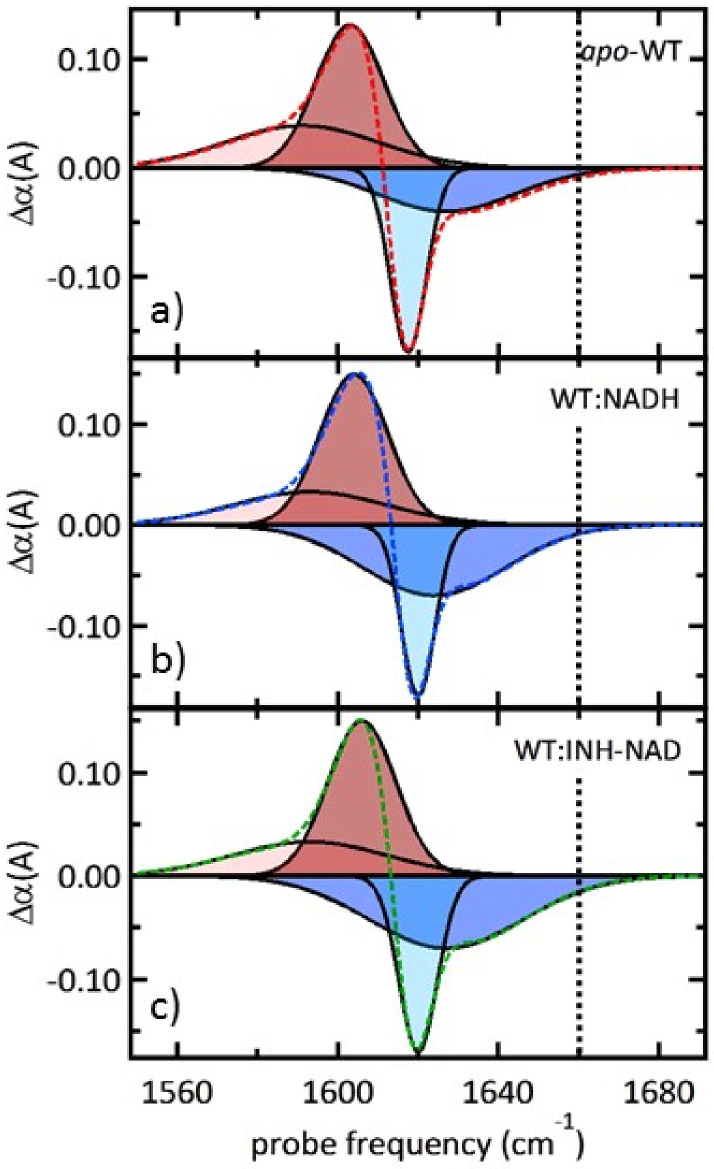

Fig. S3: Examples of fitting cross-sections through 2D-IR spectra with a pump-frequency of 1620 cm^{-1}. Figure shows fitting of cross-sections through the spectrum of wild type InhA based on the parameters listed in table S1 for a) *apo*-, b) NADH bound and c) INH-NAD inhibited samples. The regions of solid colour represent each of the individual functions used: 'low freq', pale red; 'ESA', dark red; 'bleach', pale blue and 'cross-peak', blue. The dashed lines show the sum of the components and the overall fit. The key results discussed in the text focus upon the increase in amplitude of the beach and cross peak features (pale blue and blue respectively) upon changing from apo, to NADH bound and INH-NAD inhibitor bound samples. The variation in size of the cross peak signal and its small shift to higher wavenumber, discussed in the main text, is also shown by comparing cross peak amplitudes at the point of the black dashed lines.

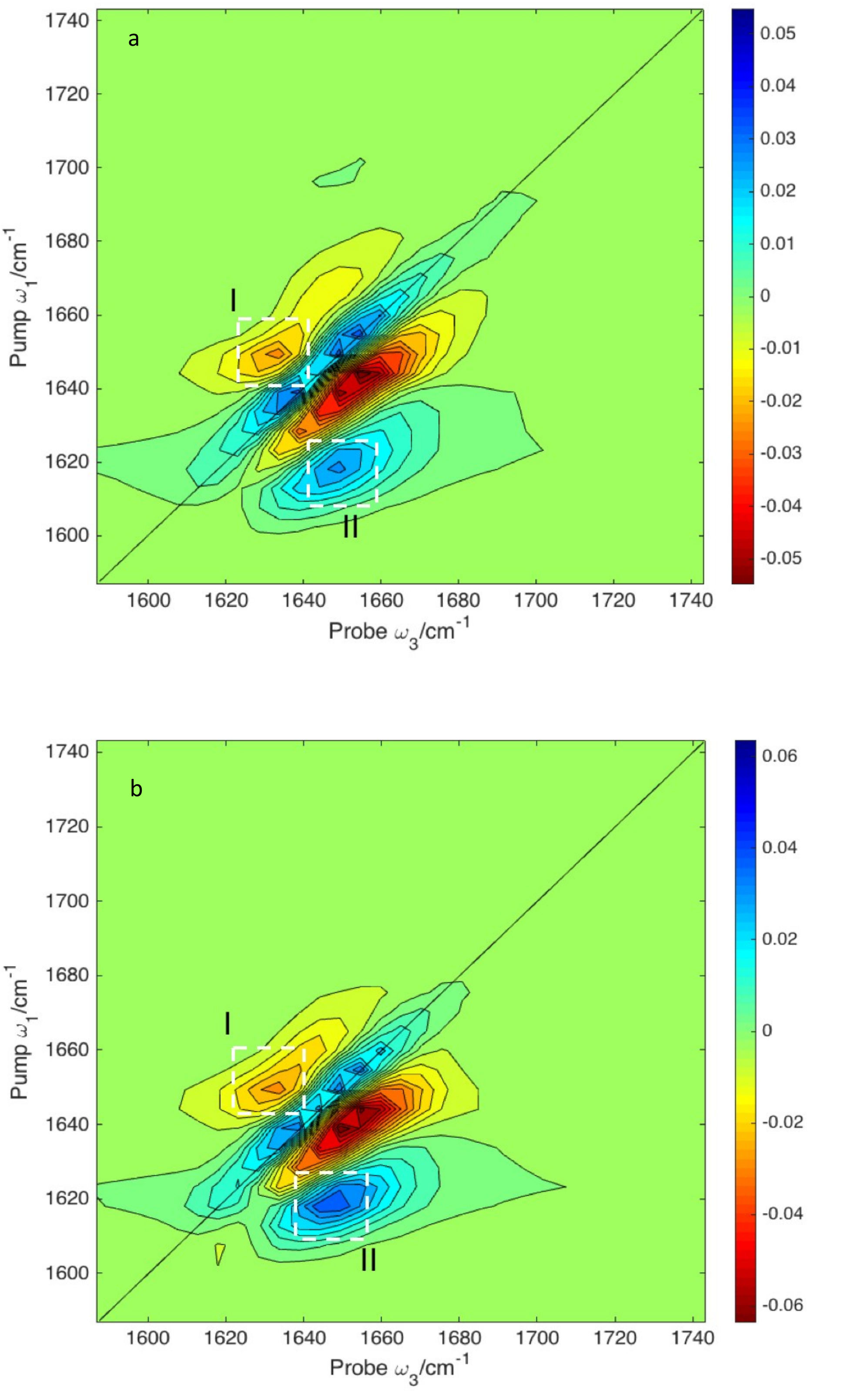

Fig S4: The result of plotting the simulated <ZZZZ>-<ZXXZ> signal for a) wild type InhA and b) the S94A mutation. This polarisation dependent 2D-IR signal has been shown to suppress diagonal peaks and enhance off-diagonal features. In this case, off-diagonal peaks linking pump-frequency of 1620 cm^{-1} to probe frequencies near 1640 cm^{-1} (see boxes I and II) provide further evidence of different coupling of β secondary structural elements in the region of the spectrum observed in experiments.

Table S1: Results of fitting cross-sections through 2D-IR spectra of wild-type InhA taken at a pump frequency of 1620 cm^{-1} to Gaussian functions. All data were normalised to the β-sheet v=0-1 transition of the apo-protein at 1620 cm^{-1}. Parameters without errors quoted were held constant based upon known values to improve quality of fitting to unknowns.

**WT**	**parameter**	***apo*-WT**	**WT:NADH**	**WT:INH-NAD**
**Low freq.**	A_0 (Δabs)	0.039 ± 0.002	0.033 ± 0.008	0.033 ± 0.006
	ω_0 (cm^{-1})	1593	1593	1593
	$\Delta\omega_0$ (cm^{-1})	27	27	27
**ESA**	A_1 (Δabs)	0.12 ± 0.01	0.17 ± 0.04	0.17 ± 0.03
	ω_1 (cm^{-1})	1606 ± 1	1607 ± 1	1609 ± 2
	$\Delta\omega_1$ (cm^{-1})	12 ± 1	13 ± 2	12 ± 2
**Bleach (β-sheet)**	A_2 (Δabs)	-0.19 ± 0.02	-0.19 ± 0.02	-0.19 ± 0.03
	ω_2 (cm^{-1})	1616.5	1618	1618
	$\Delta\omega_2$ (cm^{-1})	6.3 ± 0.2	5.9 ± 0.4	6.7 ± 0.5
**β-sheet Cross-peak**	A_3 (Δabs)	-0.04 ± 0.01	-0.07 ± 0.01	-0.08 ± 0.01
	ω_3 (cm^{-1})	1624 ± 2	1625 ± 3	1627 ± 4
	$\Delta\omega_3$ (cm^{-1})	24 ± 1	25 ± 2	27 ± 2
**Bleach ratio**	**A_3/A_2 (%)**	**21** ± 1	**37** ± 1	**42** ± 1

Table S2: Results of fitting cross-sections through 2D-IR spectra of P193A-InhA taken at a pump frequency of 1620 cm^{-1} to Gaussian functions. All data were normalised to the β-sheet v=0-1 transition of the apo-protein at 1620 cm^{-1}. Parameters without errors quoted were held constant based upon known values to improve quality of fitting to unknowns.

**P193A**	**parameter**	***apo*-P193A**	**P193A:NADH**	**P193A:INH-NAD**
**Low freq.**	A_0 (Δabs)	0.039 ± 0.007	0.042 ± 0.004	0.041 ± 0.008
	ω_0 (cm^{-1})	1593	1593	1593
	$\Delta\omega_0$ (cm^{-1})	27	27	27
**ESA**	A_1 (Δabs)	0.24 ± 0.08	0.25 ± 0.01	0.25 ± 0.02
	ω_1 (cm^{-1})	1608 ± 2	1609 ± 1	1608 ± 2
	$\Delta\omega_1$ (cm^{-1})	12 ± 2	12 ± 1	12 ± 1
**Bleach (β-sheet)**	A_2 (Δabs)	-0.35 ± 0.04	-0.36 ± 0.03	-0.35 ± 0.03
	ω_2 (cm^{-1})	1616.8	1617.2	1617.2
	$\Delta\omega_2$ (cm^{-1})	5.7 ± 0.3	5.9 ± 0.4	5.6 ± 0.4
**β-sheet Cross-peak**	A_3 (Δabs)	-0.09 ± 0.04	-0.09 ± 0.05	-0.09 ± 0.05
	ω_3 (cm^{-1})	1622 ± 2	1623 ± 2	1624 ± 2
	$\Delta\omega_3$ (cm^{-1})	21 ± 2	20 ± 2	21 ± 2
**Bleach ratio**	**A_3/A_2 (%)**	**25**± 1	**25** ± 1	**26** ± 1

Table S3: Results of fitting cross-sections through 2D-IR spectra of S94A-InhA taken at a pump frequency of 1620 cm^{-1} to Gaussian functions. All data were normalised to the β-sheet v=0-1 transition of the apo-protein at 1620 cm^{-1}. Parameters without errors quoted were held constant based upon known values to improve quality of fitting to unknowns.

**S94A**	**parameter**	***apo*-S94A**	**S94A:NADH**	**S94A:INH-NAD**
**Low freq.**	A_0 (Δabs)	0.06 ± 0.02	0.016 ± 0.005	0.021 ± 0.005
	ω_0 (cm^{-1})	1593	1593	1593
	$\Delta\omega_0$ (cm^{-1})	27	27	27
**ESA**	A_1 (Δabs)	0.09 ± 0.01	0.11 ± 0.02	0.11 ± 0.02
	ω_1 (cm^{-1})	1606 ± 1	1607 ± 2	1608 ± 2
	$\Delta\omega_1$ (cm^{-1})	12 ± 1	12 ± 2	11 ± 2
**Bleach (β-sheet)**	A_2 (Δabs)	-0.16 ± 0.01	-0.17 ± 0.02	-0.17 ± 0.03
	ω_2 (cm^{-1})	1615.5	1616.5	1617.2
	$\Delta\omega_2$ (cm^{-1})	5.2 ± 0.2	5.4 ± 0.4	5.5 ± 0.4
**β-sheet Cross-peak**	A_3 (Δabs)	-0.019 ± 0.003	-0.033 ± 0.01	-0.035 ± 0.01
	ω_3 (cm^{-1})	1624 ± 3	1625 ± 3	1626 ± 3
	$\Delta\omega_3$ (cm^{-1})	23 ± 3	25 ± 3	27 ± 3
**Bleach ratio**	**A_3/A_2 (%)**	**12** ± 1	**19** ± 1	**21** ± 1

Table S4: Results of fitting cross-sections through 2D-IR spectra of W222A-InhA taken at a pump frequency of 1620 cm^{-1} to Gaussian functions. All data were normalised to the β-sheet v=0-1 transition of the apo-protein at 1620 cm^{-1}. Parameters without errors quoted were held constant based upon known values to improve quality of fitting to unknowns.

**W222A**	**parameter**	***apo*-W222A**	**W222A:NADH**	**W222A:INH-NAD**
**Low freq.**	A_0 (Δabs)	0.050 ± 0.006	0.061 ± 0.006	0.09 ± 0.02
	ω_0 (cm^{-1})	1593	1593	1593
	$\Delta\omega_0$ (cm^{-1})	27	27	27
**ESA**	A_1 (Δabs)	0.25± 0.01	0.22 ± 0.01	0.53 ± 0.03
	ω_1 (cm^{-1})	1610 ± 2	1607 ± 1	1610 ± 1
	$\Delta\omega_1$ (cm^{-1})	14 ± 1	13 ± 1	12 ± 1
**Bleach (β-sheet)**	A_2 (Δabs)	-0.62 ± 0.09	-0.62 ± 0.04	-0.71 ± 0.07
	ω_2 (cm^{-1})	1619.4	1618	1619.4
	$\Delta\omega_2$ (cm^{-1})	5.3 ± 0.3	5.6 ± 0.2	6.1 ± 0.3
**β-sheet Cross-peak**	A_3 (Δabs)	-0.13 ± 0.04	-0.16 ± 0.07	-0.21 ± 0.05
	ω_3 (cm^{-1})	1626 ± 1	1625 ± 1	1627 ± 2
	$\Delta\omega_3$ (cm^{-1})	10 ± 1	11 ± 1	21 ± 2
**Bleach ratio**	**A_3/A_2 (%)**	**21** ± 1	**26** ± 1	**30** ± 1
